# A59 IMPACT OF ISOVALERATE SUPPLEMENTATION ON MURINE ANTIBODY RESPONSES DURING ENTERIC HELMINTH INFECTION

**DOI:** 10.1093/jcag/gwae059.059

**Published:** 2025-02-10

**Authors:** N J Norton, J M Lane, R D FitzPatrick, D M Gatti, E L Jensen, L A Reynolds

**Affiliations:** Biochemistry & Microbiology, University of Victoria, Victoria, BC, Canada; Biochemistry & Microbiology, University of Victoria, Victoria, BC, Canada; Biochemistry & Microbiology, University of Victoria, Victoria, BC, Canada; Biochemistry & Microbiology, University of Victoria, Victoria, BC, Canada; Biochemistry & Microbiology, University of Victoria, Victoria, BC, Canada; Biochemistry & Microbiology, University of Victoria, Victoria, BC, Canada

## Abstract

**Background:**

Parasitic worm (helminth) infections significantly contribute to human morbidity, with nearly one fifth of the global population chronically infected. Our lab has shown that levels of the short chain fatty acid isovalerate are increased during a murine intestinal helminth infection and that isovalerate supplementation increases helminth fecundity, implying increased worm fitness.

**Aims:**

Our research aims to elucidate the potential immunomodulatory impact of isovalerate during enteric helminth infection.

**Methods:**

Using the small intestinal-colonizing mouse roundworm, *Heligmosomoides polygyrus (Hp),* to model enteric helminth infections, we examined the influence of isovalerate supplementation in the drinking water on total and *Hp* antigen-specific antibody responses during primary (1^o^) & secondary (2^o^) *Hp* infections in both sexes of C57BL/6J mice. Circulating and mucosal antibody responses were analyzed using enzyme-linked immunosorbent assays and the impact of isovalerate supplementation on lymphocyte populations was assessed in the gut-draining lymph nodes, the spleen, and Peyer’s Patches using flow cytometry.

**Results:**

Data from one experimental repeat shows a trending reduction in total systemic and total mucosal IgA concentrations in 1^o^*Hp* infected mice that were supplemented with isovalerate, compared to mice on control drinking water. In mice supplemented with isovalerate in the 2^o^*Hp* infection model, one experimental repeat shows that isovalerate supplementation does not impact susceptibility to challenge infection or elicit changes to circulating or mucosal antibody levels.

**Conclusions:**

Preliminary data suggest that isovalerate supplementation may impact total systemic and mucosal antibody concentrations during a 1^o^*Hp* infection. Ongoing work includes solidifying our preliminary findings with experimental repeats. Future work will assess the impact of isovalerate supplementation on B cell development and antibody concentrations at steady state as well as during infection and elucidate the signalling mechanisms of isovalerate *in vivo* and *in vitro*. Furthering our understanding of how isovalerate affects the antibody response during a helminth infection may reveal novel immunomodulatory mechanisms in the intestine and may be applicable to other gut-restricted diseases.

**Funding Agencies:**

CIHRTRIANGLE

